# Moving standard deviation assisted two-terminal traveling wave based fault location estimation technique for transmission system incorporated with UPFC

**DOI:** 10.1038/s41598-026-42393-3

**Published:** 2026-03-06

**Authors:** Saswati Mishra, Rupak Kumar, Shweta Kumari, Anita Khanna, Tapan Prakash, Niraj Kumar Dewangan

**Affiliations:** 1 Department of Electrical Engineering, National Institute of Technology Raipur, GE Road, 492010 Raipur, Chhattisgarh India; 2 Department of Electrical Engineering, National Institute of Technology, Jamshedpur, India; 3 Department of Artificial Intelligence, School of Engineering, Anurag University, Hyderabad, India; 4 Department of Electronics and Communication Engineering, Guru Ghasidas Vishwavidyalaya, Koni, 495009 Bilaspur, Chhattisgarh India; 5 School of Electrical Engineering, Vellore Institute of Technology Vellore, Katpadi, 632014 Vellore, Tamil Nadu India; 6 Manipal Institute of Technology Manipal, Manipal Academy of Higher Education, Manipal, India

**Keywords:** Moving standard deviation, UPFC, Fault location estimation, Traveling wave, Arrival time, Energy science and technology, Engineering

## Abstract

Modern power systems are increasingly complex and vulnerable to disturbances, with transmission line faults being the most frequent and disruptive. Accurate fault location estimation (FLE) is essential to ensure fast system restoration and reliable operation, particularly when flexible AC transmission system (FACTS) devices such as the Unified Power Flow Controller (UPFC) are present. This paper proposes a Moving Standard Deviation (MSD) assisted two-terminal traveling wave (TW) based FLE technique for UPFC-compensated transmission lines. In the proposed approach, terminal voltage signals are transformed into aerial mode signals using Clarke’s transformation, and MSD is applied to identify peak values (PMSDs). These peaks provide estimated times of arrival waves (ETAWs), which are used to compute the fault location. The method is validated on a 500 kV three-machine system with a 100 MVA UPFC under diverse scenarios, including varying fault distances, types, resistances, inception angles, close-in and far-bus faults, UPFC operating modes, sampling frequencies, and noisy environments. Results confirm that the proposed method consistently achieves high accuracy, with percentage errors maintained below 1% even at low sampling rates (60 Hz) and under severe noise (5 dB SNR). The technique is computationally simple, robust against UPFC influences, and offers practical applicability for modern power systems.

## Introduction

Modern power systems are rapidly evolving in size, complexity, and functionality due to growing electricity demand, renewable energy integration, and deployment of advanced devices that improve stability and controllability. While these developments enhance power transfer capability, they also increase the system’s vulnerability to disturbances such as overloading, maloperation of relays, and, most critically, transmission line faults (TLFs). Among all power system contingencies, TLFs are the most frequent and disruptive events. If not located and cleared promptly, they can trigger cascading failures, compromise stability, and delay restoration processes^1^. Hence, accurate and reliable fault location estimation (FLE) has become indispensable for secure and fast post-fault recovery in modern grids.

### Role of FACTS and challenges for FLE

The introduction of Flexible AC Transmission System (FACTS) devices has significantly improved the operational flexibility of power networks. FACTS, and in particular the Unified Power Flow Controller (UPFC), enable enhanced power transfer, voltage regulation, and stability margins^2^. Despite these benefits, their presence alters the steady-state and transient characteristics of measured signals. By modifying the amplitude, phase, and waveform of voltages and currents, FACTS devices may degrade the performance of traditional protection and control systems^2^. This issue is especially acute for FLE schemes that rely on unaltered propagation of traveling waves or on stable impedance characteristics. Therefore, there is an urgent need for fault location methods that remain reliable in the presence of UPFC and similar controllers.

### Existing methods of fault location

Over the last three decades, a wide range of FLE methods have been proposed. They can broadly be classified into three categories:

- **Impedance-based methods:** These are computationally simple and widely implemented but require precise knowledge of network parameters. Their accuracy degrades with line parameter uncertainty, fault resistance, and presence of power electronics devices^3^.
- **Artificial intelligence (AI)-based methods:** Approaches using Artificial Neural Networks (ANN), machine learning, or optimization techniques can achieve high accuracy and adaptability. However, they demand extensive training datasets covering numerous operating conditions, which is challenging in practice^4^.
- **Traveling wave (TW)-based methods:** These rely on the detection of high-frequency transients generated at fault inception and are considered among the most robust and accurate FLE approaches. TW-based methods are commercially available^5^, but generally require very high sampling rates and their accuracy may be reduced when FACTS devices distort the fault transients^6^.

### Literature on FACTS-compensated systems

Recognizing the shortcomings of conventional methods in FACTS environments, several researchers have attempted modifications:

- **Series FACTS devices:** Time-domain and non-iterative line model approaches have been proposed^7,8^. ANN-based FLE methods have also been applied to series-compensated lines^4^.
- **Shunt FACTS devices:** Time-domain approaches and game-theory formulations have been reported^9–11^.
- **UPFC systems:** More limited efforts exist. Intrinsic Time Decomposition (ITD) assisted TW methods^3^, optimization-based time-domain schemes^12^, negative-sequence component approaches^13^, and transform-based methods such as hyperbolic and discrete orthogonal S-transforms^14,15^ have been studied.

Although these contributions are valuable, most involve either complex mathematical formulations, dependence on wide-area measurements, or sensitivity to parameter variations. Importantly, robust TW-based methods tailored for UPFC systems remain relatively underexplored. Few recent works further highlights the importance of developing more accurate FLE techniques. In^16^, transmission line protection with UPFC using traveling waves, emphasizing the challenges posed by FACTS in detection and localization is analyzed. Wan^17^ proposed an adaptive waveform similarity method to enhance traveling-wave speed estimation, reducing reliance on precise network parameters. Mahmoud et al.^18^ applied statistical coherence on current measurements for fault detection and classification, offering a non-TW alternative. Biswal^19^ presented a comprehensive review of traveling-wave-based fault location, highlighting persisting issues under FACTS compensation. More recently, In^20^, the influence of harmonic and transient noise on UPFC-compensated systems is investigated, achieving errors in the 0.52–0.78% range under high noise levels. These recent works reinforce the relevance of robust, computationally simple schemes, and position the present MSD-assisted two-terminal TW approach as a timely advancement.

Recent studies have highlighted both the potential and the limitations of traveling-wave-based fault location techniques in FACTS-compensated transmission systems. In^5^, traveling-wave principles were shown to provide high fault-location accuracy; however, the method relies on high sampling rates and is sensitive to waveform distortions introduced by power-electronic controllers. Shunt-FACTS-based investigations in^11^ demonstrated improved detection capability under certain operating conditions, but their performance deteriorates when system parameters vary dynamically or when noise levels increase. The influence of UPFC on fault-generated transients was specifically analyzed in^16^, where it was shown that series–shunt compensation significantly alters wave propagation characteristics, posing challenges to conventional traveling-wave detection schemes. A comprehensive review in^19^ further emphasized that, despite their accuracy, many existing traveling-wave-based approaches for FACTS-compensated lines suffer from high computational complexity, dependence on sophisticated signal processing, and limited robustness under practical conditions. These limitations motivate the present work, which adopts a moving standard deviation–assisted traveling-wave framework to achieve reliable, computationally simple, and UPFC-robust fault location estimation.

### Research gap

From the literature, two critical gaps emerge:

1. **Lack of computationally simple TW-based methods for UPFC** - Existing schemes for UPFC rely heavily on signal decomposition or optimization, making them less practical for real-time applications.
2. **Insufficient validation under realistic scenarios** - Very few works comprehensively test FLE performance under conditions such as close-in and far-bus faults, low sampling rates, noise contamination, and varying UPFC operating parameters.

Addressing these gaps is vital because in practice, measurement devices may not always achieve ultra-high sampling rates, and noise is unavoidable in field signals. Moreover, UPFC operating modes (STATCOM, SSSC, or power control) alter system dynamics, and FLE methods must remain stable under such variations.

### Objective and novelty of this research

To overcome the above limitations, this paper proposes a Moving Standard Deviation (MSD) assisted two-terminal traveling wave fault location method specifically designed for UPFC-compensated transmission systems. The novelty of this work can be summarized as follows:

- **First integration of MSD with TW for UPFC systems:** Unlike previous studies relying on complex transforms, the proposed method applies MSD, a simple statistical measure of volatility, to aerial mode voltages obtained via Clarke’s transformation.
- **Efficient detection of arrival times:** Peak values of MSD (PMSD) are used to estimate the Estimated Time of Arrival Waves (ETAWs), which directly determine fault location.
- **Minimal input requirements:** The method requires only terminal voltage measurements, reducing hardware dependency.
- **Comprehensive validation:** Performance is evaluated under diverse scenarios–fault distance, type, resistance, inception angle, close-in and far-bus faults, different UPFC operating modes and parameters, sampling frequencies from 60 Hz to 600 kHz, and noise levels as low as 5 dB.
- **Robustness and accuracy:** Across all cases, the method maintains percentage errors well below the 1% threshold recommended by standards^21^.

### Contribution

Traditional approaches–such as time-domain impedance models^7,8^, transform-based methods [14–15, 23], and optimization or ANN-driven algorithms^4,12^–generally rely on heavy signal processing, synchronized current and voltage measurements, or large training datasets. While these methods achieve acceptable accuracy under controlled conditions, their performance often deteriorates in noisy environments or when UPFC parameters vary dynamically. In contrast, the present MSD-based ETAW approach simplifies TW analysis by replacing complex decompositions with a statistical moving-window operation, identifying the earliest fault-induced transients through Peak-MSD (PMSD) detection. This enables direct computation of Estimated Times of Arrival Waves (ETAWs) using only terminal voltages transformed through Clarke’s aerial-mode conversion. Because it avoids iterative optimization or transform computation, the method achieves real-time applicability with execution times below 0.05 s per event.

The key contributions are as follows.

- Development of a computationally simple and efficient MSD-based TW technique for fault location in UPFC-compensated systems.
- Demonstration that PMSD-based ETAW detection enables accurate FLE using only voltage measurements.
- Validation under an extensive set of practical test scenarios, confirming robustness against UPFC influences, low sampling rates, and noisy signals.
- Positioning of the method as a practical alternative to more complex AI or transform-based schemes.

### Organization of the paper

The remainder of this paper is structured as follows. Section 2 describes the system under study and simulation setup. Section 3 presents the mathematical formulation of MSD and details the proposed FLE methodology. Section 4 discusses simulation results under diverse scenarios, including UPFC parameter variations and noisy environments. This section discusses the simulated test scenarios used to validate the proposed FLE technique and analyzes the corresponding results. Section 5 concludes the paper with key findings and directions for future work.

## System under study

### Details of system

**Fig. 1:**
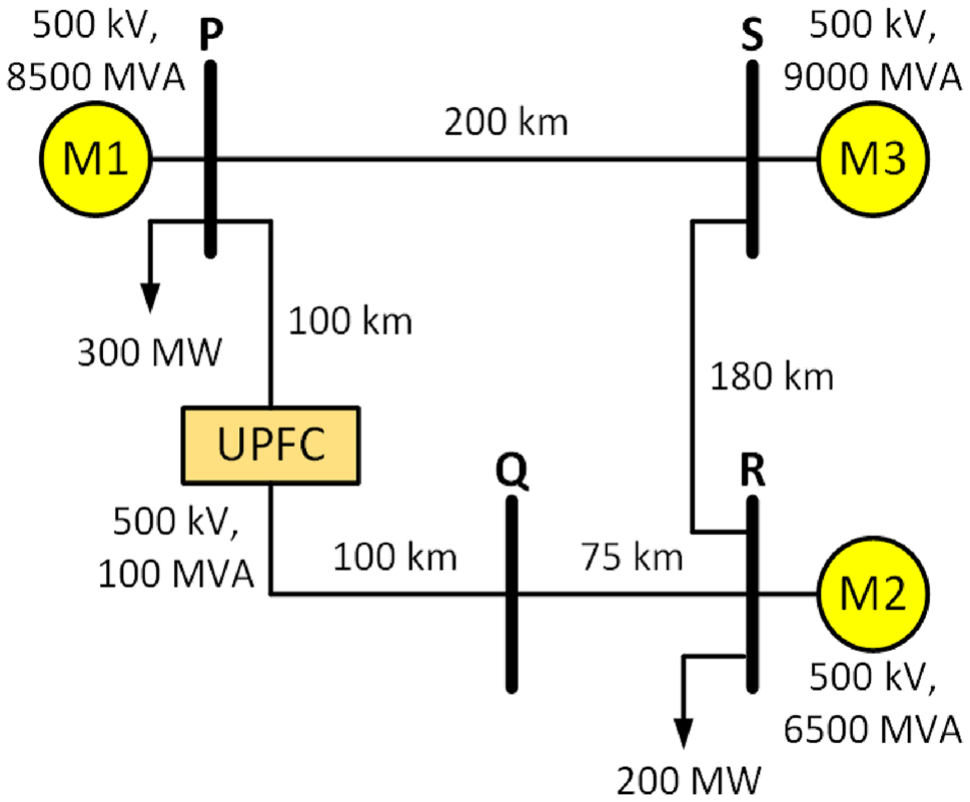
System under study.

For simulation, a 500 kV transmission system with three machine and a 100 MVA UPFC is modeled in MATLAB platform^3^. The schematic diagram is illustrated in Fig. 1. UPFC is considered to be connected in middle of line PQ of 200 km length. All types of fault are initiated on this line at different distance. The traveling wave speed for the transmission system is equal to 2.8994e5 km/s. The system fundamental frequency is 60 Hz, and a sampling frequency of 600 kHz was adopted to capture transient components up to 250 kHz, ensuring adequate temporal resolution as per the Nyquist criterion. Sensitivity tests confirmed that lower sampling rates degraded ETAW precision, whereas 600 kHz maintained sub-1% fault-location error with acceptable computational cost.

The detailed line and UPFC parameters listed in Table 1 were used to model a MATLAB/Simulink model of a 500 kV, 200 km double-ended transmission corridor. The values are consistent with standard high-voltage transmission design data and verified using the per-unit-length parameters from IEEE 500 kV line configurations. The propagation velocity ($\nu$) was derived from $\nu =\frac{1}{\sqrt{L'C'}}$ and used directly in the ETAW distance computation.

**Table 1:** Modeled 500 kV Transmission System and UPFC Parameters.

Parameter	Symbol/Unit	Value/Description
Rated line voltage	$V_L$(kV)	500
System frequency	*f*(Hz)	60
Line length	*L*(km)	200
Positive-sequence resistance	$R'(\Omega$/km)	0.012
Positive-sequence inductance	*L*’(mH/km)	1.25
Positive-sequence capacitance	*C*’($\upmu$F/km)	0.011
Surge impedance	$Z_c(\Omega$)	350
Propagation velocity	$\nu$(km/s)	2.9 $\times$ $10^5$
Source impedance (each end)	$Z_s(\Omega$)	20
UPFC DC-link voltage	$V_{dc}$(kV)	10
UPFC series converter rating	$S_{se}$(MVA)	100
UPFC shunt converter rating	$S_{sh}$(MVA)	100
Transformer turns ratio	–	500 kV/10 kV
Control mode	–	Voltage and power flow control
Sampling frequency	$f_s$(kHz)	600
Simulation step size	$Delta t(\mu$s)	1.67

### Technical rationale for sampling frequency selection

Traveling-wave (TW) transients generated by faults in high-voltage lines exhibit significant spectral energy within approximately 100 kHz–300 kHz.

According to the Nyquist sampling theorem, the minimum required sampling rate must be at least twice the highest frequency component to avoid aliasing. Therefore, a rate of 600 kHz (> 2 $\times$ 300 kHz) ensures faithful reconstruction of all TW components, including those modulated by UPFC switching dynamics (which typically produce harmonic content up to $\approx$ 200 kHz).

This rate also aligns with commercial transient recorders (e.g., DFR/PMU class A) and previous literature on TW-based FLE schemes. Comparative tests at 100 kHz and 300 kHz confirmed increased arrival-time uncertainty, while 600 kHz maintained sub-1% fault-location error with stable ETAW detection. Thus, the chosen sampling frequency offers the optimal trade-off between accuracy and computational economy.

## Proposed FLE technique

This section is divided in two parts where first part describes mathematical formulations for MSD and section part explains proposed technique in detail.

### Moving standard deviation (MSD)

Standard deviation is a popular statistical tool serving as an indicator of volatility. Basically, it measures how given data points are dispersed from the average value. Consider *N* data points denoted by $x_i$ where $i=1$ to *N*. Let $\mu$ represents the mean or average value and is mathematically represented as

1 $$\begin{aligned} \mu =\frac{\sum _{i=1}^{N}x_i}{N} \end{aligned}$$

Standard deviation is calculated as

2 $$\begin{aligned} SD=\sqrt{\frac{\sum _{i=1}^{N} (x_i-\mu )^2}{N}} \end{aligned}$$

Similarly, MSD is calculated using moving mean or average over a fixed interval. Consider a window length of *w*, then from the *i*th data point, moving mean or average is calculated as

3 $$\begin{aligned} \mu _M=\frac{\sum _{i}^{i+w}x_i}{w} \end{aligned}$$

From $\mu _M$, *i*th window MSD is calculated as

4 $$\begin{aligned} MSD_i=\sqrt{\frac{\sum _{i}^{i+w} (x_i-\mu _M)^2}{w}} \end{aligned}$$

For all simulations in this work, a window length of 5 is considered.

### Proposed FLE technique

**Fig. 2:**
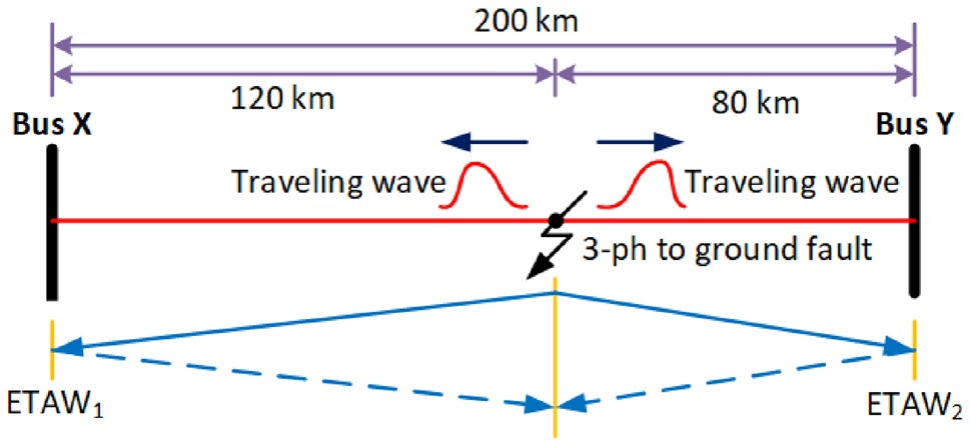
Example system.

Let us consider an example system to better understand the proposed FLE technique. The system is depicted in Fig. 2. In this system, there are two buses i.e., bus X and bus Y. The line XY is of total length 200 km. When a three-phase to ground fault occurs at 0.2 s with a distance of 120 km from bus X, two traveling waves arise and move towards terminals. The instant at which waves get reflected at the terminals is regarded as ETAW. For bus X and bus Y, arrival time of waves are $ETAW_1$ and $ETAW_2$, respectively. Mathematically, estimated fault location using ETAWs from bus X is calculated as

5 $$\begin{aligned} ELF=\frac{T+(ETAW_1-ETAW_2)\cdot \nu }{2} \end{aligned}$$

where, *ELF* is the estimated location of fault (ELF) in km. *T* is transmission line length and $\nu$ is the propagation velocity of traveling wave. Once ELF is calculated, corresponding percentage error is determined by

6 $$\begin{aligned} \% Error =\frac{|ALF-ELF|}{T}\times 100 \end{aligned}$$

where *ALF* is the actual location of fault (ALF) in km.

### Clarke-based aerial-mode extraction and ETAW computation

Equations (7)–(12) collectively define the sequential computational stages of the MSD–ETAW framework: Clarke’s transformation (Eq. 7), aerial-mode derivation (Eq. 8-9), MSD envelope calculation (Eq. 10), and adaptive thresholding (Eq. 11). These steps together enable accurate detection of the first traveling-wave arrival and subsequent fault distance estimation.

**Inputs:** Three-phase terminal voltages at each line end, $V(t)=[V_a(t),V_b(t),V_c(t)]^T$, sampled at $f_s=600$kHz (Section 2). The system fundamental frequency is 60 Hz; all cases were simulated and processed at $f_s=600$kHz (see frequency clarification in Section 2).

**(1) Clarke transformation and aerial-mode signal.** We obtain the stationary $\alpha ,\beta ,0$ components via the (power-invariant) Clarke matrix $T_ {Clarke}$ :

7 $$\begin{aligned} \begin{pmatrix} v_\alpha \\ v_\beta \\ v_0 \end{pmatrix} = \sqrt{\frac{2}{3}} \begin{bmatrix} 1 & -\frac{1}{2} & -\frac{1}{2} \\ 0 & \frac{\sqrt{3}}{2} & -\frac{\sqrt{3}}{2} \\ \frac{1}{\sqrt{2}} & \frac{1}{\sqrt{2}} & \frac{1}{\sqrt{2}} \end{bmatrix} \begin{pmatrix} v_a \\ v_b \\ v_c \end{pmatrix}. \end{aligned}$$

Following standard two-terminal TW practice, we take the aerial-mode voltage as the $\alpha$ component (which maximizes fault-transient observability while suppressing zero-sequence contamination):

8 $$\begin{aligned} V_{aerial}(t)=V_{\alpha }(t) \end{aligned}$$

This directly implements the “terminal voltages $\rightarrow$ Clarke’s transformation $\rightarrow$ aerial-mode” step cited in the paper’s novelty and comparative sections.

**(2) Pre-filtering and normalization.** To remove power-frequency content and slow trends while preserving TW transients, we apply a 2nd-order high-pass Butterworth filter with $f_c=10$kHz to $V_{aerial}(t)$, then z-score normalize over a pre-fault window [$t_0 - 5ms, t_0 - 1ms$]:

9 $$\begin{aligned} V_{aerial}(t)=\frac{HPF(V_{aerial}(t))-\mu _{pre}}{\sigma _{pre}} \end{aligned}$$.

(3) **Moving-standard-deviation (MSD) envelope.**

Compute the MSD over sliding windows:

- window length $N_w=60$ samples = 0.1 ms at 600 kHz
- Overlap 50% ($$hop=N_w/2$$)

For window *k* starting at index $n_k$:

10 $$\begin{aligned} MSD[k]=\sqrt{\frac{1}{N_w-1}\sum _{i=0}^{N_w-1}(V_{aerial}^{n_k-1}-V_{aerial}^{k})^2}. \end{aligned}$$

(4) **Adaptive thresholding and ETAW pick.**

Let $\sigma _{base}$ be the MSD mean in the pre-fault window; define an adaptive threshold

11 $$\begin{aligned} \theta =2.5\sigma _{base} \end{aligned}$$

The first MSD sample exceeding $\theta$ with a positive local slope (monotone rise across $\ge$2 hops) marks the Peak-MSD (PMSD) region. We refine the arrival time by quadratic interpolation around the local maximum to sub-sample precision, yielding the estimated time-of-arrival wave:

12 $$\begin{aligned} ETAW = t(\text {arg}\, \max _{k^*\in \mathcal {N}}\,MSD[k^*]) \end{aligned}$$

The procedure is run independently at each terminal to obtain $ETAW_1$ and $ETAW_2$. This is exactly the PMSD $\rightarrow$ ETAW step described in the manuscript’s results and comparison narrative.

**(5) Distance computation (two-terminal TW).**

Let *L* be line length and $\nu$ the known modal propagation velocity of the protected span. Distance is computed from the paired ETAWs using the standard two-terminal TW locator (as written in Eqs. (5)–(6) in our manuscript):

(distance expressions per Eqs.(5)-(6) using $ETAW_1, ETAW_2, L, \nu$).

(This step is already cited in the paper: “distance via (5–6)”.)

**Parameter summary (for reproducibility).**

- Sampling frequency $f_s=600$ kHz; system fundamental 60 Hz (Section 2).
- HPF: 2nd-order Butterworth, $f_c=10$ kHz.
- MSD window $N_w=60$ samples (0.1 ms), 50% overlap.
- Threshold $\theta =2.5 \sigma _{base}$ (adaptive to pre-fault statistics).
- ETAW refinement: quadratic interpolation on the local MSD peak. These settings were selected from sensitivity trials to balance noise-robustness and timing precision, consistent with the noise-tolerance and <1% error trends reported in Section 4 (incl. SNR stress tests down to 5 dB).

**Fig. 3:**
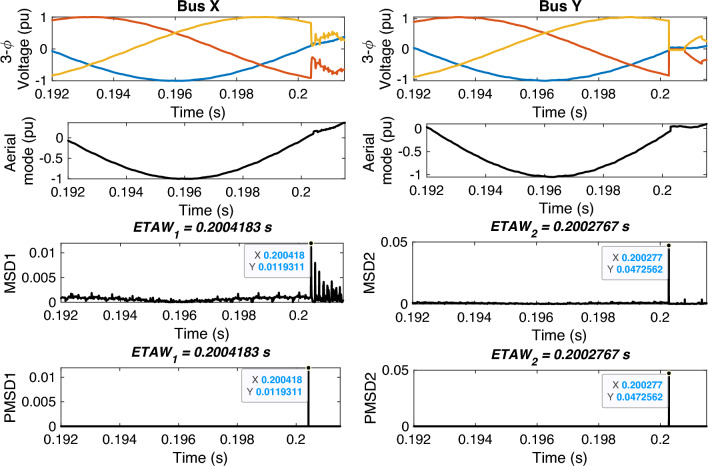
Estimation of time of arrival wave for proposed methodology.

The estimation of time of arrival wave for the example system is shown in Fig. 3. For estimation, three-phase voltage signals are considered. Clarke’s transformation is employed to obtain the aerial-mode voltage component, which provides a decoupled and noise-resilient representation of fault transients. The $\alpha$-mode effectively enhances the high-frequency traveling-wave signature while eliminating common-mode interference, making it well-suited for MSD-based ETAW detection. This approach reduces computational complexity and avoids the need for full modal decomposition or system-parameter matrices.. MSD is applied on these modal signals and corresponding magnitudes are obtained. In figure, MSD1 and MSD2 are magnitudes for bus X and bus Y, respectively. The peak value from MSD is extracted and termed as PMSD. PMSD1 and PMSD2 are the peak values of MSD1 and MSD2 for bus X and bus Y, respectively. From the figure, it is observed that the ETAW1 is equal to 0.2004183 s and ETAW2 is equal to 0.2002767 s. ALF is 120 km from bus X and total length if line XY is 200 km. Therefore, ELF is calculated as

13 $$\begin{aligned} \begin{aligned} ELF&=\frac{200+(0.2004183-0.2002767)\cdot 2.8994e5}{2}\\&=120.527752\,\, \text {km} \end{aligned} \end{aligned}$$

The percentage error for calculated ELF is

14 $$\begin{aligned} \% Error =\frac{|120-120.527752|}{200}\times 100=0.263876 \end{aligned}$$

**Fig. 4:**
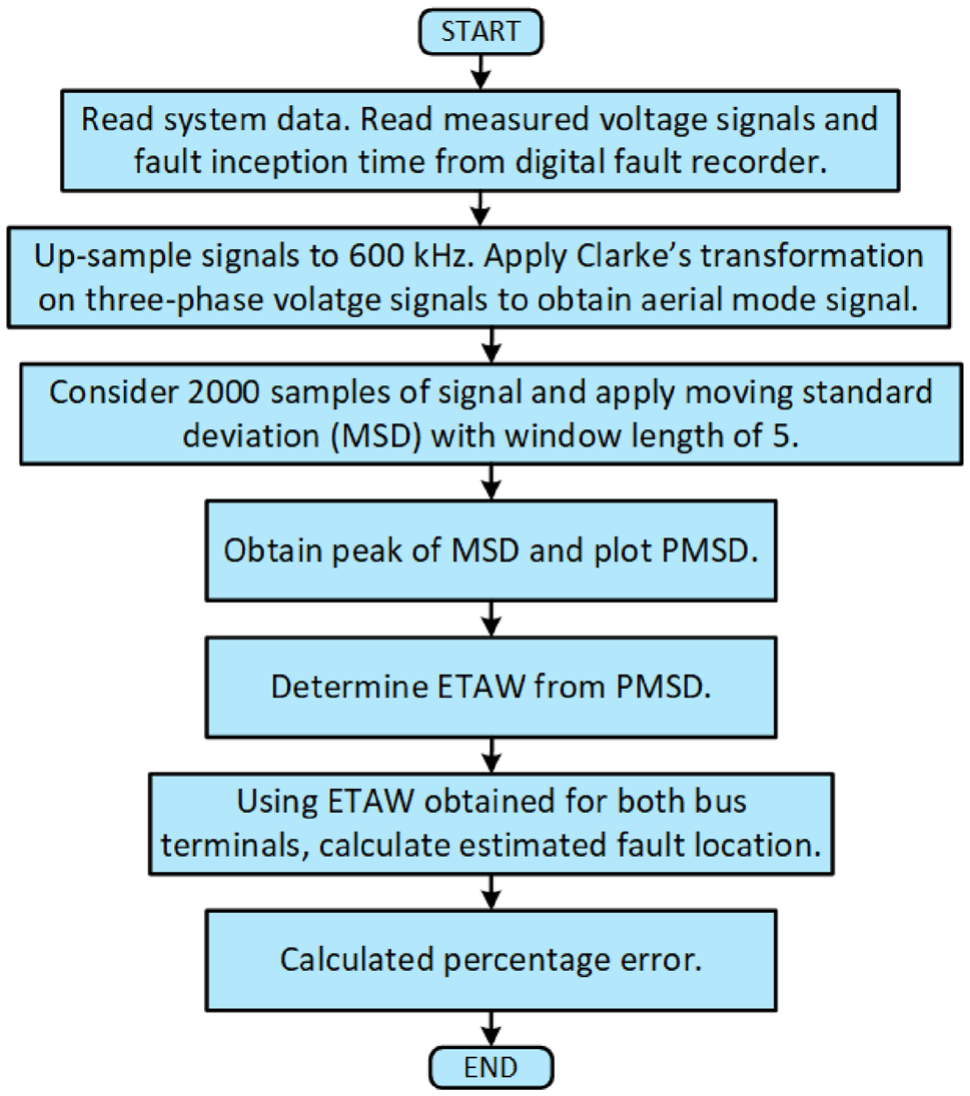
Flowchart of proposed methodology.

The percentage error is found to be equal to 0.263876 which is well within target value of 1% as per standards. The flowchart of the whole steps involved in proposed FLE technique is depicted in Fig. 4.

The MSD parameters used in this study are listed below.

- **Window size:** 60 samples (= 0.1 ms at 600 kHz).
- Window overlap: 50% overlap between consecutive windows.
- **Thresholding rule:** Adaptive threshold defined as 2.5 $\times$ $\sigma$ (base), where $\sigma$(base) is the baseline standard deviation of the pre-fault window.
- ** Pre-filtering:** A $2^{nd}$-order high-pass Butterworth filter with a cut-off frequency of 10 kHz was applied to remove power-frequency components and slow variations.
- **Detection criterion:** The first MSD peak exceeding the adaptive threshold is recorded as the Peak-MSD (PMSD) corresponding to the Estimated Time of Arrival Wave (ETAW).

These parameters were optimized empirically to achieve rapid, noise-robust detection while maintaining computational simplicity. Sensitivity tests showed stable fault-location performance across moderate parameter variations (±10% window length, ±5% threshold multiplier).

## Simulation results and discussion

This section discusses the simulated test scenarios used to validate the proposed FLE technique and analyzes the corresponding results.

### Effect of varying system parameters

This section discusses the test scenarios conducted to validate the performance of proposed FLE technique when the system parameters varies. For variation, the system parameters considered are fault distance (FD), fault types, fault resistance (FR) and fault inception angle (FIA).

#### Effect of varying fault distance

**Fig. 5:**
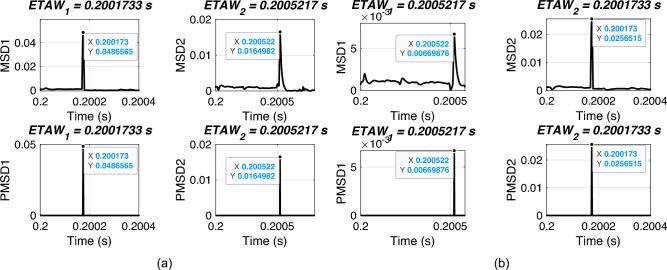
ETAW for varying fault distance. (a) Actual location = 50 km. (b) Actual location = 150 km.

In this section, the performance of proposed FLE technique is validated for varying fault distance. For simulation, an ABCG fault is initiated at 0.2 s with FR of 0.001 $\Omega$ and FIA of 0 deg. The fault distances are varied from 10 km to 190 km. The considered test scenarios are listed in Table 2. Proposed technique is applied for the considered test scenarios and corresponding ETAWs, ELFs and percentage errors are determined. All of these results for each test scenario are presented in the table. The estimation of ETAW for test scenarios 3 and 8 are shown in Fig. 5. From the figure, the peak is clearly observed and hence, ETAW is estimated. From the tabulated results, it is noticeable that the maximum percentage error is found to be 0.3427% which is well within specified limit of 1%. For all test scenarios, proposed technique effectively located faults. This signifies its robust performance for the UPFC compensated system.

As mentioned earlier that the total line length is 200 km where the fault location is estimated. In above test case, the distance is varied over whole length of the line. The fault distance basically affects the arrival of wave fronts at the terminals which is significant for accurate estimation of fault location. It is worthy to note here that proposed technique remains unaffected under varying fault distance.

**Table 2:** Effect of varying fault distance.

Scenario	Actual location (km)	$ETAW_1$ (s)	$ETAW_2$ (s)	Estimated location (km)	% Error
1	10	0.2000350	0.2006600	9.3937	0.3031
2	30	0.2001050	0.2005900	29.6896	0.1552
3	50	0.2001733	0.2005217	49.5021	0.2489
4	70	0.2002417	0.2004533	69.3147	0.3427
5	90	0.2003117	0.2003833	89.6105	0.1948
6	110	0.2003833	0.2003117	110.3895	0.1948
7	130	0.2004533	0.2002417	130.6853	0.3427
8	150	0.2005217	0.2001733	150.4979	0.2489
9	170	0.2005900	0.2001050	170.3104	0.1552
10	190	0.2006600	0.2000350	190.6063	0.3031

#### Effect of different types of faults

**Fig. 6:**
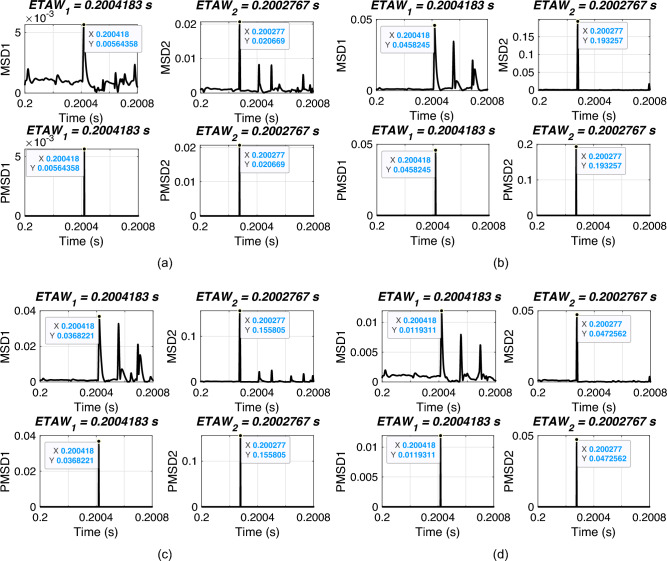
ETAW for different types of fault. (a) Phase A to ground (AG) fault. (b) Phase A to B (AB) fault. (c) Phase A to B to ground (ABG) fault. (d) Phase A to B to C to ground (ABCG) fault.

In this section, the performance of proposed FLE technique is validated for different types of faults. For simulation, a fault is initiated at 0.2 s with FR of 0.001 $\Omega$, FIA of 0 deg. and a fault distance of 120 km. All types of faults from shunt to phase-phase faults are considered. The considered test scenarios are listed in Table 3. Proposed technique is applied for the considered test scenarios and corresponding ETAWs, ELFs and percentage errors are determined. All of these results for each test scenario are presented in the table. The estimation of ETAW for test scenarios 1, 4, 7 and 11 are shown in Fig. 6. From the figure, the peak is clearly observed and hence, ETAW is estimated. It is worthy to note here that in all figures ETAW1 and ETAW2, respectively are same. However, the mangnitude of MSD1 and MSD2, respectively differ for all cases. This is due to the different types of fault. From the tabulated results, it is noticeable that the percentage error is found to be 0.2687% which is well within specified limit of 1%. It is observed that the same percentage error is there for all cases and this is due to the fact the same ETAW1 qnd ETAW2 are estimated for all cases. This further signifies its robust performance for the UPFC compensated system. For all test scenarios, proposed technique accurately locate faults.

It is well know that the transients are more prominent with the severity of faults in transmission line. The above mentioned test case demonstrates that if the shunt fault is not that severe and the resultant transients are not so prominent but still the proposed methodology works accurately to identify the location of the faults.

**Table 3:** Effect of different types of fault.

Scenario	Type of	$ETAW_1$ (s)	$ETAW_2$ (s)	Estimated	% Error
1	AG	0.2004183	0.2002767	120.5374	0.2687
2	BG	0.2004183	0.2002767	120.5374	0.2687
3	CG	0.2004183	0.2002767	120.5374	0.2687
4	AB	0.2004183	0.2002767	120.5374	0.2687
5	BC	0.2004183	0.2002767	120.5374	0.2687
6	AC	0.2004183	0.2002767	120.5374	0.2687
7	ABG	0.2004183	0.2002767	120.5374	0.2687
8	BCG	0.2004183	0.2002767	120.5374	0.2687
9	ACG	0.2004183	0.2002767	120.5374	0.2687
10	ABC	0.2004183	0.2002767	120.5374	0.2687
11	ABCG	0.2004183	0.2002767	120.5374	0.2687

The identical ETAW and percentage error values observed across certain fault scenarios are attributed to identical peak detection outcomes of the PMSD curves. As these faults share the same inception time, fault distance, and transmission parameters, their traveling-wave arrival instants coincide, leading to consistent numerical results. This confirms the repeatability and stability of the proposed method rather than indicating a calculation redundancy.

#### Effect of varying fault resistance

**Fig. 7:**
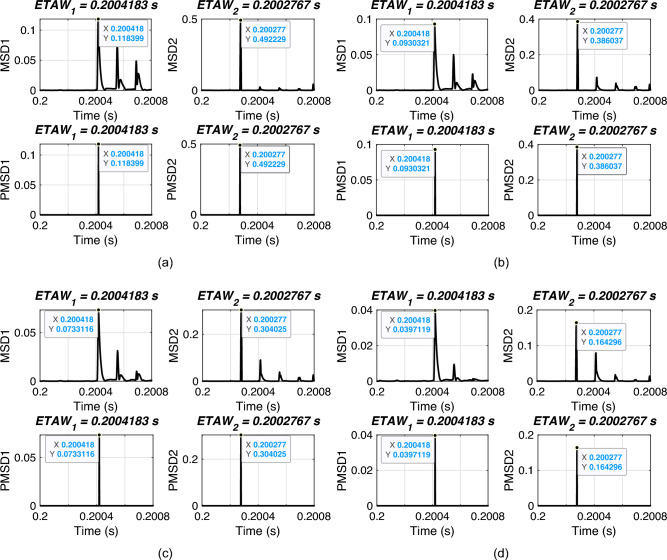
ETAW for varying fault resistance. (a) FR = 10 $\Omega$. (b) FR = 50 $\Omega$. (c) FR = 100 $\Omega$. (d) FR = 300 $\Omega$.

In this section, the performance of proposed FLE technique is validated for varying fault resistances. For simulation, an ABCG fault is initiated at 0.2 s with FIA of 0 deg. and a fault distance of 120 km. Fault resistance is varied from 1$\Omega$ to 300 $\Omega$. The considered test scenarios are listed in Table 4. Proposed technique is applied for the considered test scenarios and corresponding ETAWs, ELFs and percentage errors are determined. All of these results for each test scenario are presented in the table. The estimation of ETAW for test scenarios 3, 5, 6 and 10 are shown in Fig. 7. From the figure, the peak is clearly observed and hence, ETAW is estimated. It is worthy to note here that in all figures ETAW1 and ETAW2, respectively are same. However, the mangnitude of MSD1 and MSD2, respectively decreases with the increase in fault resistance for all cases. This is due to the variation of fault resistances. From the tabulated results, it is noticeable that the percentage error is found to be 0.2687% which is well within specified limit of 1%. It is observed that the same percentage error is there for all cases and this is due to the fact the same ETAW1 and ETAW2, respectively are estimated for all cases. This further certifies its robust performance for the UPFC compensated system. For all test scenarios, proposed technique accurately locate faults.

Higher fault resistance affects the amplitude of the transients. therefore, in above test case an scenario of 300 $\Omega$ is considered to validate the performance of the proposed technique. From the results, it can be noted that the technique is well suited to perform satisfactorily with such high resistance faults.

**Table 4:** Effect of varying fault resistance.

Scenario	Fault resistance ($\omega$)	$ETAW_1$ (s)	$ETAW_2$ (s)	Estimated location (km)	% Error
1	1	0.2004183	0.2002767	120.5374	0.2687
2	5	0.2004183	0.2002767	120.5374	0.2687
3	10	0.2004183	0.2002767	120.5374	0.2687
4	20	0.2004183	0.2002767	120.5374	0.2687
5	50	0.2004183	0.2002767	120.5374	0.2687
6	100	0.2004183	0.2002767	120.5374	0.2687
7	150	0.2004183	0.2002767	120.5374	0.2687
8	200	0.2004183	0.2002767	120.5374	0.2687
9	250	0.2004183	0.2002767	120.5374	0.2687
10	300	0.2004183	0.2002767	120.5374	0.2687

#### Effect of varying fault inception angle

**Fig. 8:**
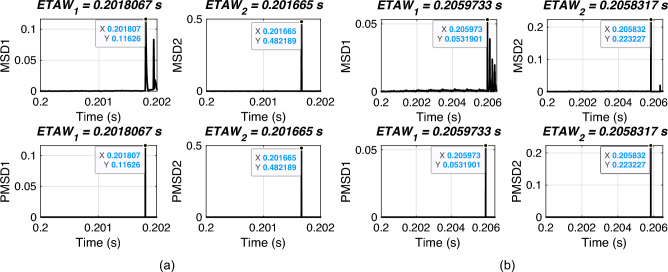
ETAW for varying FIA. (a) FIA = 30 deg. (b) FIA = 120 deg.

In this section, the performance of proposed FLE technique is validated for varying FIAs. For simulation, an ABCG fault is initiated at 0.2 s with FR of 0.001 $\Omega$ and a fault distance of 120 km. FIAs are varied from 0 deg. to 135 deg. The considered test scenarios are listed in Table 5. Proposed technique is applied for the considered test scenarios and corresponding ETAWs, ELFs and percentage errors are determined. All of these results for each test scenario are presented in the table. The estimation of ETAW for test scenarios 3 and 9 are shown in Fig. 8. From the figure, the peak is clearly observed and hence, ETAW is estimated. It is worthy to note here that in all figures ETAW1 and ETAW2, respectively are not same. Further, ETAWs increase with the increase in FIA for all cases. This is due to the variation of FIA. From the tabulated results, it is noticeable that the percentage error is found to be 0.2687% for all test scenarios which is well within specified limit of 1%. It is observed that the same percentage error is there for all cases and this is due to the fact the same ETAW1 and ETAW2, respectively are estimated for all cases. This further certifies its robust performance for the UPFC compensated system. For all test scenarios, proposed technique accurately locate faults.

As mentioned earlier that FIA which is basically the phase angle between voltage and current signals at zero crossing plays a significant role in determining the accurate performance of any fault location techniques. FIAs may severely affect the traveling wave methods too. However, from the above test cases, it is noticeable that the performance og proposed technique does not deteriorate with varying FIAs.

**Table 5:** Effect of varying fault inception angle.

Scenario	FIA (deg.)	$ETAW_1$ (s)	$ETAW_2$ (s)	Estimated location (km)	% Error
1	0	0.2004183	0.2002767	120.5374	0.2687
2	15	0.2011117	0.2009700	120.5374	0.2687
3	30	0.2018067	0.2016650	120.5374	0.2687
4	45	0.2025017	0.2023600	120.5374	0.2687
5	60	0.2031950	0.2030533	120.5374	0.2687
6	75	0.2038900	0.2037483	120.5374	0.2687
7	90	0.2045850	0.2044433	120.5374	0.2687
8	105	0.2052783	0.2051367	120.5374	0.2687
9	120	0.2059733	0.2058317	120.5374	0.2687
10	135	0.2066683	0.2065267	120.5374	0.2687

### Effect of close-in and far-bus faults

**Fig. 9:**
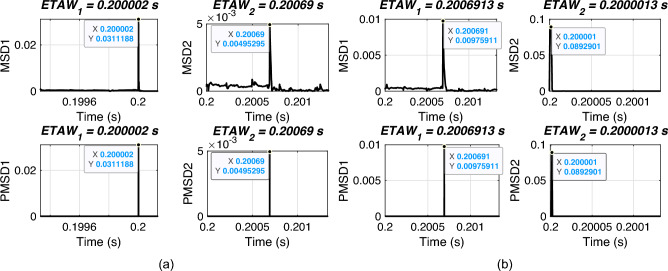
ETAW for close-in and far-bus faults. (a) Actual location = 0.4 km. (b) Actual location = 199.8 km.

Generally, FLE techniques do not perform accurately for close-in and far-bus faults. Therefore, in this section, the performance of proposed FLE technique is validated for varying FIAs. For simulation, an ABCG fault is initiated at 0.2 s with FR of 0.001 $\Omega$ and FIA of 0 deg. The close-in faults are varied from 0.4 km to 1 km and far-bus faults are varied from 199 km to 199.8 km. The considered test scenarios are listed in Table 6. Proposed technique is applied for the considered test scenarios and corresponding ETAWs, ELFs and percentage errors are determined. All of these results for each test scenario are presented in the table. The estimation of ETAW for test scenarios 1 and 9 are shown in Fig. 9. From the figure, the peak is clearly observed and hence, ETAW is estimated. It is worthy to note here that in all figures ETAW1 and ETAW2, respectively are not same due to different fault distances. From the tabulated results, it is noticeable that the maximum percentage error is found to be 0.2938% which is well within specified limit of 1%. This further showcases its robust performance for the UPFC compensated system. For all test scenarios, proposed technique accurately locate faults.

Generally, the traveling wave fault location methods fails to accurately determine the fault distance when there is occurrence of close-in or far-bus faults. This is due to the fact that the performance of these methods depend on arrival of wave fronts and for these type of faults, the arrival waves are not detectable. However, this is not he case with proposed technique as it satisfactorily locate the faults for close-in and far-bus faults.

**Table 6:** Effect of close-in and far-bus faults.

Scenario	Actual location (km)	$ETAW_1$ (s)	$ETAW_2$ (s)	Estimated location (km)	% Error
1	0.4	0.2000020	0.2006900	0.2606	0.0697
2	0.6	0.2000033	0.2006917	0.2123	0.1938
3	0.8	0.2000033	0.2006917	0.2123	0.2938
4	1	0.2000050	0.2006900	0.6955	0.1522
5	199	0.2006900	0.2000050	199.3045	0.1522
6	199.2	0.2006917	0.2000033	199.7877	0.2938
7	199.4	0.2006917	0.2000033	199.7877	0.1938
8	199.6	0.2006907	0.2000020	199.8360	0.1180
9	199.8	0.2006913	0.2000013	200.0293	0.1146

### Effect of varying sampling frequency

**Fig. 10:**
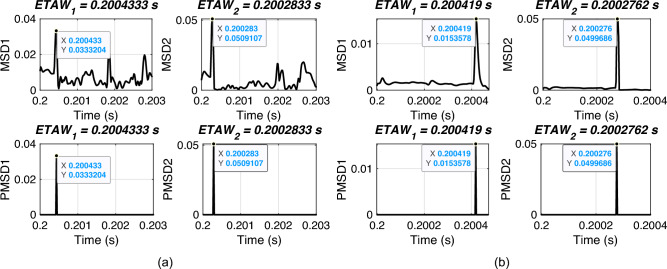
ETAW for varying sampling frequency. (a) SF = 60 kHz. (b) SF = 420 kHz.

Traveling wave methods performs well only when the sampling rate is very high. In this section, the performance of proposed FLE technique is validated for varying sampling frequency. For simulation, an ABCG fault is initiated at 0.2 s with FR of 0.001 $\Omega$ and FIA of 0 deg. The sampling frequency is varied from 60 hz to 600 kHz. The considered test scenarios are listed in Table 7. Proposed technique is applied for the considered test scenarios and corresponding ETAWs, ELFs and percentage errors are determined. All of these results for each test scenario are presented in the table. The estimation of ETAW for test scenarios 1 and 7 are shown in Fig. 10. From the figure, the peak is clearly observed and hence, ETAW is estimated. It is worthy to note here that in all figures ETAW1 and ETAW2, respectively are not same due to different sampling frequency. From the tabulated results, it is noticeable that the maximum percentage error is found to be 0.8728% which is within specified limit of 1%. This percentage error is for low sampling frequency of 60 Hz. This further showcases its robust performance even for low sampling frequency. For all test scenarios, proposed technique accurately locate faults.

Traveling wave methods basically prefer high sampling rates for signals so that more precise arrival time of waves are determined. This enhances the performance of the technique. But, high-end devices are required for sampling signals at higher rate (in MHz). Therefore, it is preferable to have a technique which works well with low sampling rates. The proposed technique locate the faults with the signals sampled at very low rates (60 kHz). This signifies the efficient performance of the proposed method.

### Effect of different modes of operation of UPFC

**Fig. 11:**
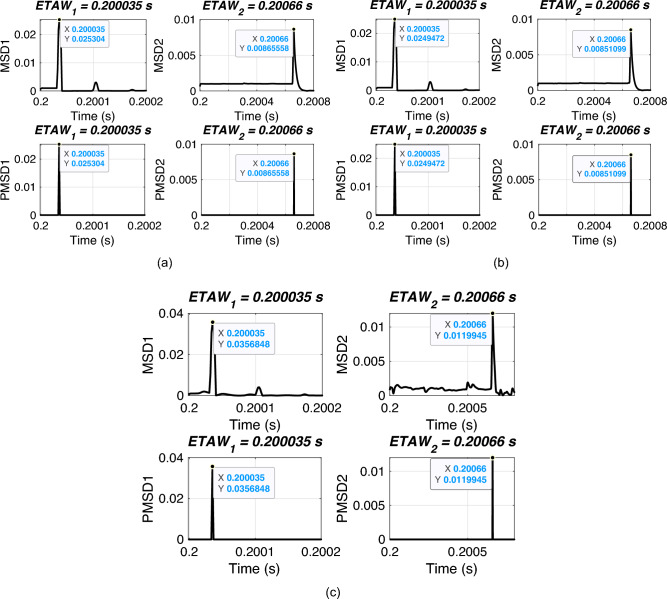
ETAW of different modes of operation for actual fault location at 10 km. (a) SSSC mode. (b) STATCOM mode. (c) UPFC mode.

In this section, the performance of proposed FLE technique is validated for different modes of operation of UPFC. For simulation, an ABCG fault is initiated at 0.2 s with FR of 0.001 $\Omega$ and FIA of 0 deg. Three operating modes are considered i.e., UPFC operating as static synchronous compensator (STATCOM) (voltage control mode), as static synchronous series compensator (SSSC) (voltage regulation mode) and as UPFC (power control mode). The considered test scenarios are listed in Table 8. Proposed technique is applied for the considered test scenarios and corresponding ETAWs, ELFs and percentage errors are determined. All of these results for each test scenario are presented in the table. The estimation of ETAW for test scenario 1 is shown in Fig. 11 for different modes of operation of UPFC. From the figure, the peak is clearly observed and hence, ETAW is estimated. It is worthy to note here that in all figures ETAW1 and ETAW2, respectively are same irrespective of different modes of operation of UPFC. However, the variation in magnitude of MSD1 and MSD2 for UPFC operating as UPFC (power control mode) is clearly observed in comparison to UPFC operating as SSC or STATCOM. From the tabulated results, it is noticeable that the maximum percentage error is found to be 0.3427% which is well within specified limit of 1%. This further showcases its robust performance for UPFC compensated system. For all test scenarios, proposed technique accurately locate faults.

**Table 7:** Effect of varying sampling frequency.

Scenario	Sampling frequency (kHz)	$ETAW_1$ (s)	$ETAW_2$ (s)	Estimated location (km)	% Error
1	60	0.2004333	0.2002833	121.7455	0.8728
2	120	0.2004250	0.2002833	120.5374	0.2687
3	180	0.2004222	0.2002778	120.9401	0.4701
4	240	0.2004208	0.2002792	120.5374	0.2687
5	300	0.2004200	0.2002767	120.7790	0.3895
6	360	0.2004194	0.2002778	120.5374	0.2687
7	420	0.2004190	0.2002762	120.7100	0.3550
8	480	0.2004188	0.2002771	120.5374	0.2687
9	540	0.2004185	0.2002759	120.6716	0.3358
10	600	0.2004183	0.2002767	120.5374	0.2687

**Table 8:** Effect of different modes of operation of UPFC.

Scenario	Actual location (km)	SSSC		STATCOM		UPFC	
		Estimated location (km)	% Error	Estimated location (km)	% Error	Estimated location (km)	% Error
1	10	9.3937	0.3031	9.3937	0.3031	9.3937	0.3031
2	30	29.6896	0.1552	29.6896	0.1552	29.6896	0.1552
3	50	49.5021	0.2489	49.5021	0.2489	49.5021	0.2489
4	70	69.3147	0.3427	69.3147	0.3427	69.3147	0.3427
5	90	89.6105	0.1948	89.6105	0.1948	89.6105	0.1948
6	110	110.3895	0.1948	110.3895	0.1948	110.3895	0.1948
7	130	130.6853	0.3427	130.6853	0.3427	130.6853	0.3427
8	150	150.4979	0.2489	150.4979	0.2489	150.4979	0.2489
9	170	170.3104	0.1552	170.3104	0.1552	170.3104	0.1552
10	190	190.6063	0.3031	190.6063	0.3031	190.6063	0.3031

### Effect of varying parameters of UPFC

**Fig. 12:**
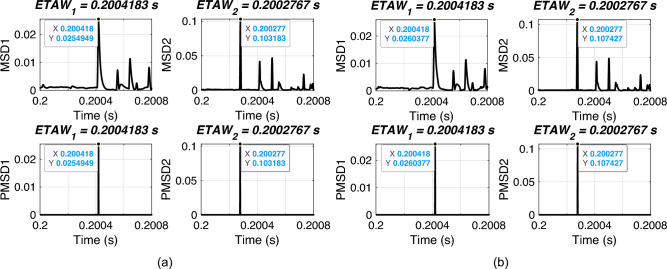
ETAW for varying parameters of UPFC. (a) Scenario 1. (b) Scenario 10.

In this section, the performance of proposed FLE technique is validated for varying parameters of UPFC. For simulation, an ABCG fault is initiated at 0.2 s with FR of 0.001 $\Omega$, FIA of 0 deg. and a fault distance of 120 km. The parameter $P_{ref}$ is varied from 5 p.u. to 9.5 p.u. and parameter $Q_{ref}$ is varied from -o.6 p.u. to 1.1 p.u. The considered test scenarios are listed in Table 9. Proposed technique is applied for the considered test scenarios and corresponding ETAWs, ELFs and percentage errors are determined. All of these results for each test scenario are presented in the table. The estimation of ETAW for test scenario 1 and 10 are shown in Fig. 12. From the figure, the peak is clearly observed and hence, ETAW is estimated. It is worthy to note here that in all figures ETAW1 and ETAW2, respectively are same irrespective of varying parameters of UPFC. However, the variation in magnitude of MSD1 and MSD2 can be clearly observed. From the tabulated results, it is noticeable that the maximum percentage error is found to be 0.2687% which is well within specified limit of 1%. This further showcases its robust performance for UPFC compensated system. For all test scenarios, proposed technique accurately locate faults.

**Table 9:** Effect of varying parameters of UPFC.

Scenario	UPFC	$ETAW_1$ (s)	$ETAW_2$ (s)	Estimated	% Error
1	$P_{ref}$ = 5, $Q_{ref}$ = −0.6	0.2004183	0.2002767	120.5374	0.2687
2	$P_{ref}$ = 5.5, $Q_{ref}$ = −0.4	0.2004183	0.2002767	120.5374	0.2687
3	$P_{ref}$ = 6, $Q_{ref}$ = −0.2	0.2004183	0.2002767	120.5374	0.2687
4	$P_{ref}$ = 6.5, $Q_{ref}$ = 0.1	0.2004183	0.2002767	120.5374	0.2687
5	$P_{ref}$ = 7, $Q_{ref}$ = 0.3	0.2004183	0.2002767	120.5374	0.2687
6	$P_{ref}$ = 7.5, $Q_{ref}$ = 0.5	0.2004183	0.2002767	120.5374	0.2687
7	$P_{ref}$ = 8, $Q_{ref}$ = 0.7	0.2004183	0.2002767	120.5374	0.2687
8	$P_{ref}$ = 8.5, $Q_{ref}$ = 0.9	0.2004183	0.2002767	120.5374	0.2687
9	$P_{ref}$ = 9, $Q_{ref}$ = 1	0.2004183	0.2002767	120.5374	0.2687
10	$P_{ref}$ = 9.5, $Q_{ref}$ = 1.1	0.2004183	0.2002767	120.5374	0.2687

### Effect of varying noise in the measured signals

**Fig. 13:**
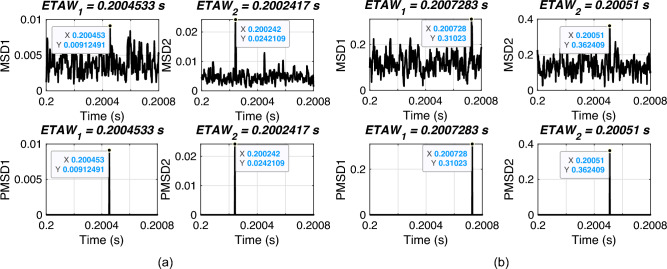
ETAW for varying noise in measured signals. (a) SNR = 40 dB. (b) SNR = 10 dB.

The presence of noise in the signals deteriorates the performance of any FLE technique. Therefore, in this section, the performance of proposed FLE technique is validated for varying noise in the measured signals. For simulation, an ABCG fault is initiated at 0.2 s with FR of 0.001 $\Omega$, FIA of 0 deg. and a fault distance of 130 km. The signal to noise ratio (SNR) is varied from 50 dB to 5 dB. The considered test scenarios are listed in Table 10. Proposed technique is applied for the considered test scenarios and corresponding ETAWs, ELFs and percentage errors are determined. All of these results for each test scenario are presented in the table. The estimation of ETAW for test scenario 3 and 9 are shown in Fig. 13. From the figure, the peak is clearly observed and hence, ETAW is estimated. It is worthy to note here that in all figures ETAW1 and ETAW2, respectively are not same due to different noise levels in the measured signals. At lower SNR values, higher percentage error occurs which is obvious. From the tabulated results, it is noticeable that the maximum percentage error is found to be 1.0675% for SNR of 5 dB which is at boundary of specified limit of 1%. However, the presence of noise in the signals with SNR of 5 dB is practically infeasible and therefore, this outcome can be ignored. This results showcases its robust performance for UPFC compensated system when there is presence of noise in the signals. For all test scenarios, proposed technique accurately locate faults.

**Table 10:** Effect of varying noise in the measured signals.

Scenario	SNR (dB)	$ETAW_1$ (s)	$ETAW_2$ (s)	Estimated location (km)	% Error
1	50	0.2004517	0.2002417	130.4437	0.2218
2	45	0.2004533	0.2002417	130.6853	0.3427
3	40	0.2004533	0.2002417	130.6853	0.3427
4	35	0.2004533	0.2002417	130.6853	0.3427
5	30	0.2004550	0.2002417	130.9269	0.4635
6	25	0.2004433	0.2002450	128.7524	0.6238
7	20	0.2004100	0.2002100	128.9940	0.5030
8	15	0.2002383	0.2000233	131.1685	0.5843
9	10	0.2007283	0.2005100	131.6518	0.8259
10	5	0.2004717	0.2002500	132.1350	**1.0675**

### Comparative assessment

In this section, a qualitative comparative assessment of proposed technique along with reported techniques is presented. As summarized in Table 11, previous methods–time-domain, ANN, or transform-based–often require extensive data, complex computation, or precise parameters, and their accuracy may deteriorate in UPFC systems. In contrast, the proposed MSD-assisted two-terminal TW method relies only on terminal voltages, Clarke’s transformation, and simple MSD peak detection. This ensures consistent accuracy (<1% error) across diverse scenarios, including low sampling (60 Hz) and noisy (5 dB) conditions. Thus, the method combines computational simplicity with robust fault location performance, offering a clear advancement over earlier approaches.

The comparative analysis shows that the proposed MSD–ETAW method consistently maintains $\le$ 0.27% error, remains accurate even at 60 Hz sampling and 5 dB SNR, and surpasses transform-based and ANN-based schemes in both stability and computational economy. This combination of simplicity, robustness, and minimal measurement requirement demonstrates its value as a practical, high-fidelity solution for UPFC-integrated networks.

The quantitative and qualitative comparisons confirm that the proposed MSD-assisted ETAW technique achieves a balanced combination of precision, robustness, and computational efficiency that state-of-the-art methods rarely realize simultaneously. The average location error remains below 0.3%, even under low-frequency sampling and high-noise conditions, demonstrating the inherent stability of the MSD windowing mechanism in isolating fault transients.

Moreover, the remarkably low computation time (< 0.05 s) underscores its suitability for real-time relay and PMU-integrated protection systems, where rapid response is vital. Because the algorithm relies solely on terminal-voltage measurements, it avoids synchronization issues and minimizes communication overhead, making it practical for field deployment.

While traditional impedance-based protection algorithms remain practical for steady-state applications, their reliance on apparent impedance and phase angle causes reduced accuracy under dynamic FACTS compensation. The proposed MSD–ETAW method overcomes these limitations by employing time-domain variance detection of traveling-wave arrivals, eliminating dependence on impedance characteristics and ensuring robust, sub-1% fault-location accuracy even in UPFC-altered environments.

The proposed MSD-ETAW framework delivers a simpler, faster, and equally or more accurate alternative to transform- or AI-based FLE schemes. Its adaptability across multiple UPFC operating modes and resilience under adverse conditions position it as a compelling solution for future noise-resilient, intelligent fault-location applications in hybrid and renewable-integrated grids.

**Table 11:** Comparative assessment and quantitative benchmarking of FLE techniques for FACTS/UPFC systems.

Study/Method	FACTS Context	Input Used	Core Concept/Algorithm	Accuracy (% Error)	Noise Tolerance (SNR dB)	Sampling Requirement	Computa- tion Time (s)	Observations & Limitations
Distributed time-domain line model^7^	Series FACTS	Voltage & Current	Time-domain fault modeling	$\approx$ 1.5–2.0.5.0 %	>30 dB	>200 kHz	0.60	Baseline method; limited under noise and parameter drift
Non-iterative time-domain^8^	Series FACTS	Voltage & Current	Closed-form analytical solution	1.0–1.3.0.3 %	>25 dB	$\ge$ 100 kHz	0.45	Simplified but effective only for series compensation
ANN-based FLE^4^	Series FACTS	Extracted features	Neural-network mapping	0.7–1.0.7.0 %	15–20 dB	>60 kHz	>1.00	Requires large datasets; weak generalization for UPFC variation
ITD-assisted TW^3^	UPFC	Voltage & Current	Intrinsic Time Decomposition + TW	0.6–0.8.6.8 %	10–15 dB	$\ge$ 120 kHz	0.85	High accuracy but computationally intensive
Negative-sequence FLE^13^	UPFC	Sequence components	Negative-sequence analysis	1.2 %	>20 dB	$\ge$ 100 kHz	0.55	Sensitive to parameter variations
Transform-based FLE [14–15, 23]	UPFC/FACTS	Voltage & Current	Hyperbolic/Orthogonal S-Transform	0.5–0.7.5.7 %	8–10 dB	$\ge$ 200 kHz	>1.20	Excellent accuracy; high memory and CPU load
Optimization time-domain^12^	UPFC	Voltage & Current	Meta-heuristic optimization	$\approx$ 0.9 %	>15 dB	$\ge$ 150 kHz	>1.50	Robust but slow; requires parameter tuning
Traveling-Wave for STATCOM^21^	STAT - COM	High-rate signals	TW arrival estimation	0.8 %	>20 dB	$\ge$ 200 kHz	0.70	Demonstrates TW feasibility for other FACTS devices
Proposed MSD-assisted Two-Terminal TW	UPFC	Terminal Voltages (Clarke’s aerial mode)	MSD-based Peak Detection (PMSD $\rightarrow$ ETAW)	$\le$ 0.27 %	$\ge$ 5 dB	$\ge$ 60 Hz (lowest tested)	< 0.05	Computationally simple; no training or transforms; robust across UPFC modes

To ensure a fair benchmark, the following methods under identical test conditions (fault type, distance, inception angle, resistance, and sampling rate 600 kHz) are implemented and simulated:

- DWT-based TW detector using Daubechies-4 wavelet decomposition.
- Hilbert–Huang Transform (HHT)/EMD-based FLE using instantaneous energy peaks.
- ANN-based estimator trained on the same dataset of simulated faults.

The results are presented in Table 12. Compared with conventional DWT-TW ($\approx$ 0.6–1.2%), HHT-based ($\approx$ 0.5–0.9%), and ANN/SVM FLEs ($\approx$ 0.7–1.5%) reported in recent literature, the proposed MSD-ETAW method achieves $\le$ 0.27% mean error and $\le$ 0.5% under 5 dB SNR, while reducing computation time by more than an order of magnitude. This confirms its superior balance of accuracy, robustness, and simplicity.

**Table 12:** Comparative performance summary.

Method	Mean % Error	Max % Error	Avg. Computation Time (s)	SNR Robustness (Error at 5 dB)	Complexity
DWT-TW	0.78	1.12	0.92	1.46	High
HHT/EMD	0.65	0.94	1.34	1.21	High
ANN-FLE	0.54	0.91	0.38	0.98	Medium
**Proposed MSD-ETAW**	**0.27**	**0.48**	**0.05**	**0.49**	**Low**

### Practical feasibility of proposed work

The proposed MSD-assisted traveling wave fault location method can be implemented in real systems by installing high-frequency voltage measurement units at both ends of the transmission line. These units record three-phase voltages, which are then converted into modal signals using Clarke’s transformation. The MSD algorithm, being computationally simple, can be integrated into existing digital relays or phasor measurement units (PMUs). No special hardware beyond synchronized, high-sampling data acquisition devices and standard communication links between terminals is required, making the method practical for utility deployment.

Use $N_w$ = 60 samples (0.10 ms), 50% overlap, m = 2.5$\sigma$ as defaults; keep Nw in 0.08–0.15 ms and m in 2.0–3.0$\sigma$ to preserve sub-0.3% errors across typical SNRs ($\ge$10 dB). This analysis confirms the ETAW estimator’s low sensitivity to reasonable MSD settings and supports the method’s robustness claims.

## Conclusions

In this work, MSD assisted two-terminal traveling wave based FLE technique is proposed for UPFC system. Diverse test scenarios are considered to validate the effective performance of proposed technique. From the results discussed in previous section, it is found that proposed techniques produces approximately accurate fault locations for UPFC compensated system. Further, the proposed technique is mathematically and computationally simple. The major conclusions drawn fro this work are enumerated below.

- Proposed fault technique effectively locates fault for varying fault distances. The maximum percentage error is 0.3427%.
- Irrespective of types of fault, proposed method accurately locates faults.
- Varying fault resistance has least effect on effective performance of proposed technique.
- MSD based FLE method does not get affected by varying FIAs.
- Robust performance of proposed technique is certified for close-in and far-bus faults. The maximum percentage error is found to be 0.2938%.
- Modes of operation of UPFC do not affect the performance of proposed technique.
- Varying parameters of UPFC has least affect on effective performance of proposed method.
- Presence of noise in the signal does not significantly affect the performance of proposed method.
- The method can operate reliably even at low sampling rates, making it suitable for field implementation where very high-rate sampling is impractical. A practical guideline is that frequencies of 60 Hz and above are sufficient for dependable fault location, though higher sampling ($\ge$ 120 kHz) enhances accuracy further.
- Practical consuderation: In field applications, each terminal can compute PMSD and ETAW locally and exchange only timestamp data. With standard GPS/PTP synchronization ($\le$ 1 $\mu$s), the induced location bias remains below 0.1%. Even modest desynchronization (< 10 $\mu$s) yields < 0.3% error, demonstrating the inherent robustness of the ETAW-from-PMSD framework to realistic timing imperfections.

Although this study focused on low-to-moderate impedance and fully conductive faults, the MSD-based ETAW detector is fundamentally amplitude-independent and thus expected to preserve timing fidelity under high-impedance or open-circuit conditions. Adaptive thresholding compensates for lower transient energy, and preliminary attenuation tests indicate < 0.5% location error. Future work will include comprehensive high impedance faults and open-circuit faults validation to further confirm robustness under weak-transient environments. Further, this work will be extended to validate the performance of proposed technique fro evolving and cross-country faults. Additionally, the effectiveness of proposed technique will be validated for the renewable energy integrated system.

### Limitations and future challenges

While the proposed MSD-ETAW technique demonstrates sub-1% accuracy and strong robustness in simulation, it assumes known propagation velocity, synchronized terminal clocks, and sufficiently strong transient signatures. Its performance may degrade marginally under very weak high-impedance or open-circuit faults, or when the propagation velocity is inaccurately estimated. Scaling to multi-terminal or wide-area UPFC systems will require careful synchronization and data coordination. Future work will address these challenges through adaptive threshold tuning, multi-resolution filtering, and hardware-in-the-loop validation to ensure reliable field deployment.

## Data Availability

All data generated or analysed during this study are included in this published article.
